# Dopamine versus norepinephrine as the first-line vasopressor in the treatment of cardiogenic shock

**DOI:** 10.1371/journal.pone.0277087

**Published:** 2022-11-03

**Authors:** Soo Jin Na, Jeong Hoon Yang, Ryoung-Eun Ko, Chi Ryang Chung, Yang Hyun Cho, Ki Hong Choi, Darae Kim, Taek Kyu Park, Joo Myung Lee, Young Bin Song, Jin-Oh Choi, Joo-Yong Hahn, Seung-Hyuk Choi, Hyeon-Cheol Gwon

**Affiliations:** 1 Department of Critical Care Medicine, Samsung Medical Center, Sungkyunkwan University School of Medicine, Seoul, Republic of Korea; 2 Division of Cardiology, Department of Medicine, Heart Vascular Stroke Institute, Samsung Medical Center, Sungkyunkwan University School of Medicine, Seoul, Republic of Korea; 3 Department of Thoracic and Cardiovascular Surgery, Samsung Medical Center, Sungkyunkwan University School of Medicine, Seoul, Republic of Korea; University of Luebeck, GERMANY

## Abstract

**Background:**

Only a few observational studies using small patient samples and one subgroup analysis have compared norepinephrine and dopamine for the treatment of cardiogenic shock (CS). The objective of the present study was to investigate whether the use of norepinephrine was associated with improvements in clinical outcomes in CS patients compared to dopamine.

**Methods:**

We retrospectively reviewed hospital medical records of patients who were admitted to cardiac intensive care unit from 2012 to 2018. We included 520 patients with CS in this analysis. The primary outcome was in-hospital mortality, and serial hemodynamic data were also assessed.

**Results:**

As a first-line vasopressor, dopamine was used in 156 patients (30%) and norepinephrine in 364 patients (70%). Overall, the norepinephrine group had significantly higher severity of shock, arrest at presentation, vital signs, and lactic acid than did the dopamine group at the time of vasopressor initiation. Nevertheless, in the norepinephrine group, additional vasopressor was required in 123 patients (33.8%), which was a significantly smaller percentage than the 92 patients (56.4%) in the dopamine group who required additional vasopressor (p < 0.001). There was no significant difference in in-hospital mortality between the two groups (26.9% and 31.9%, respectively, p = 0.26). In addition, the incidence of arrhythmia was not different between the two groups (atrial fibrillation, 12.2% vs. 15.7%, p = 0.30; ventricular tachyarrhythmia, 19.9% vs. 25.3%, p = 0.18).

**Conclusions:**

The use of norepinephrine as a first-line vasopressor was not associated with reductions of in-hospital mortality or arrythmia but could reduce use of additional vasopressors in CS patients.

## Introduction

Cardiogenic shock (CS) is an ineffective cardiac output state primarily caused by cardiac dysfunction resulting in insufficient end-organ perfusion, with a high mortality rate of 40–50% [1–6]. Vasoactive drugs play a vital role in the management of patients with CS to stabilize hemodynamic status and restore adequate tissue perfusion [7, 8]. Among several vasoactive drugs, dopamine and norepinephrine are commonly used in the treatment of CS [9]. Although these two agents have different pharmacological effects, robust treatment recommendations regarding which drug is the optimal first-line vasopressor in CS have not yet been established because studies that directly compare the effects of these drugs on clinical outcomes are sparse [10].

Recently published American Heart Association (AHA) guidelines suggest that norepinephrine is the vasopressor of choice in most patients with CS [1]. However, it also noted a lack of evidence to support this conclusion and stated that dopamine can be considered as a first-line vasopressor when the patient has a low heart rate due to the arrhythmogenic effect of dopamine. Theoretically, the use of dopamine in patients with lower heart rates is expected to have a beneficial effect in restoring tissue perfusion by increasing cardiac output through chronotropic effects [11].

Therefore, we investigated whether norepinephrine was associated with improvements in clinical outcomes compared to dopamine in patients with CS overall and among various subgroups and evaluated serial hemodynamic changes and patterns regarding requirements for additional vasopressors according to use of dopamine or norepinephrine as the first-line vasopressor.

## Methods

### Study population

We retrospectively reviewed 4,659 consecutive patients who were admitted to the cardiac intensive care unit (CICU) at Samsung Medical Center from January 1, 2012, to December 31, 2018. Patients were included if they were 18 years of age or older and presented with CS regardless of etiology. CS was defined as follows: (1) inotrope or vasopressor support was required to maintain systolic blood pressure> 90 mmHg, and (2) accompanying tissue hypoperfusion was represented by serum lactate levels ≥ 2.0 mmol/L. If a patient was admitted multiple times to the CICU, we included only their first CICU admission in the analysis. A total of 767 patients met the criteria during study period. Patients who did not use dopamine and norepinephrine, who started using dopamine and norepinephrine at the same time, and who used drugs other than dopamine or norepinephrine as the first vasoactive drug were excluded. We also excluded patients with durations of vasopressor use of less than 2 hours to avoid cases of transient circulatory shock caused by vasodilatory effects caused by sedative drugs or vasovagal response without cardiogenic shock in clinical practice. Patients with shock related to bradycardia in which the use of dopamine is suggested, and acute aortic syndrome in which the role of pharmacological treatment is limited, were judged not suitable for comparing the effects of dopamine and norepinephrine and were excluded from the analysis (S1 Fig). Eligible patients were divided into dopamine and norepinephrine groups, depending on the first-line vasoactive drug used. The institutional review board of the Samsung Medical Center approved this study and waived the requirement for informed consent because of the observational nature of the research. Additionally, patient information was anonymized and de-identified prior to analysis.

### Monitoring and management of CS

Our CICU is a level 1 intensive care unit with invasive and noninvasive devices that can monitor the hemodynamic status of patients and provide advanced therapeutic technologies to support the cardiovascular system, such as intra-aortic balloon pump (IABP), extracorporeal membrane oxygenation (ECMO), and ventricular assistance devices, as well as mechanical ventilators and machines for continuous renal replacement therapy (CRRT). The details of our CICU setting are described in previous publications [12, 13].

Although there were no predefined protocols for monitoring and management of patients with CS, routine practice was based on general guidelines and publications regarding treatment of underlying cardiovascular disease and critically ill patients. Vital signs including blood pressure, heart rate, respiratory rate, and electrocardiography were continuously monitored. Arterial catheters were used to measure blood pressure. Fluid intake and output were counted hourly by a nurse. Arterial lactate was measured at the time of CS diagnosis, and then serially measured to assess response to treatment. The lactate measurement interval was determined by the attending physician. Central venous catheter or pulmonary artery catheter placement for hemodynamic monitoring was not routinely performed. To diagnose cardiovascular disease causing CS and assess cardiac function, electrocardiograms, cardiac enzymes, and echocardiography were evaluated, and additional tests such as coronary angiography were considered according to the initial test results [14].

The prescriptions for initiation, type, dose-escalation, and combinations of vasoactive drugs were left to the discretion of the physician in charge, but in general, medications were adjusted with the initial goal of achieving systolic blood pressure > 90 mmHg or mean arterial pressure > 65mmHg, and the subsequent target blood pressure was modified based on improvement of tissue perfusion. In patients with persistent shock refractory to vasoactive drugs, mechanical circulatory support devices such as IABP or ECMO were considered unless contraindicated.

### Data collection and clinical outcomes

Clinical, laboratory, and outcome data were retrospectively collected by a trained study coordinator by reviewing hospital records.

Demographic data, including age, sex, comorbidities, history of cardiac arrest, diagnosis, presence of arrhythmia, and sequential organ failure assessment (SOFA) score were recorded at admission to the CICU. To compare the effects of dopamine and norepinephrine on shock reversal and occurrence of tachycardia, data for vital signs, lactic acid, and types and doses of vasoactive drugs administered to the patient were obtained at the time of diagnosis of CS (H_0_) and 6 (H_6_), 12 (H_12_), 24 (H_24_), and 48 (H_48_) hours after that time point. In this study, we defined the time of diagnosis of CS as the time when the first-line vasoactive drug was started. Arterial blood pressure and heart rate were automatically recorded in an electronic medical record system every minute. The times of initiation, discontinuation, and dose adjustment of each drug with doses of mcg/kg/min, except for vasopressin with a dose of unit/min, were recorded by the bedside nurse. In order to quantify the level of total pharmacologic support, we used the Vasopressor Inotrope Score (VIS) as described by Gaies et al. [15]. The VIS was calculated as: dopamine dose (mcg/kg/min) + dobutamine dose (mcg/kg/min) + 100 x epinephrine dose (mcg/kg/min) + 10 x milrinone dose (mcg/kg/min) + 10,000 x vasopressin dose (unit/kg/min) + 100 x norepinephrine dose (mcg/kg/min).

The primary outcome was in-hospital mortality. Secondary outcomes included CICU mortality, duration of vasoactive drug, adverse events during vasoactive agent use, need for mechanical circulatory support devices, and need for mechanical ventilation and renal replacement therapy.

### Statistical analysis

Categorical data are presented as numbers and percentages and continuous variables are described as medians with interquartile ranges. To compare characteristics and clinical outcomes between the two groups, we used χ2 tests or Fisher’s exact tests for categorical variables, when applicable, and Mann-Whitney U tests for continuous variables. Logistic regression analysis was performed to estimate the efficacy of dopamine or norepinephrine as the first-line vasoactive agent for preventing in-hospital mortality. Multivariable logistic regression models were constructed using all variables with p-values < 0.1 from univariate analyses, and variables were potentially relevant. The results are reported as odds ratio (OR) of each variable with 95% confidence interval (CI). We conducted further analyses to assess the consistency of effects across subgroups. Subgroups were specified according to baseline variables with p-values < 0.05 for between-group comparison. Continuous variables were converted into binary variables for defining subgroups using median values. We performed a sensitivity analysis using propensity score matching to reduce the effects of potential confounders and selection bias. Propensity scores were estimated using a logistic regression model of baseline characteristics including age, sex, SOFA scores, cardiac arrest prior to CICU admission, comorbidities, diagnosis, initial rhythm, mean arterial pressure, heart rate, lactic acid, troponin I, NT-proBNP, left ventricular ejection fraction, and treatments during CICU stay. The patients were matched 1:1 by propensity scores with a caliper size of 0.9. For all analyses, a two-tailed test with a p-value less than 0.05 was considered statistically significant. Statistical analyses were performed using STATA version 16.0 (Stata Corp, College Station, TX, USA).

## Results

### Study population

Of 520 eligible patients with CS, 156 were treated with dopamine and 364 were treated with norepinephrine as the first-line vasopressor (S1 Fig). Age, sex, and comorbidities were not significantly different between the two groups (Table 1). History of cardiac arrest prior to CICU admission was more common (35.2% vs. 21.8%, p = 0.003) and the median SOFA score was higher (9 vs. 7, p = 0.03) in the norepinephrine group than in the dopamine group. Overall, acute coronary syndrome was the most common etiology of CS (44.0%) followed by acute heart failure (41.7%). In addition, there were no differences in the initial heart rhythm, level of cardiac enzyme, or echocardiographic measurements between the two groups. Baseline characteristics and in-hospital management are presented for propensity-matched patients (S1 Table).

**Table 1:** Baseline characteristics and in-hospital management.

Variables	Dopamine group (n = 156)	Norepinephrine group (n = 364)	p-value
Age, years	66 (56–77)	67 (58–77)	0.66
Male	95 (60.9)	225 (61.8)	0.84
SOFA score	7 (5–10)	9 (6–11)	0.03
Cardiac arrest prior to admission	34 (21.8)	128 (35.2)	0.003
Comorbidities			
Coronary artery disease	41 (26.3)	110 (30.2)	0.37
Chronic heart failure	47 (30.1)	122 (33.5)	0.45
Chronic lung disease	9 (5.8)	31 (8.5)	0.28
Chronic kidney disease	33 (21.1)	73 (20.1)	0.81
Chronic liver disease	6 (3.9)	19 (5.2)	0.50
Diagnosis			0.02
Acute coronary syndrome	83 (53.2)	146 (40.1)	
Heart failure	56 (35.9)	161 (44.2)	
Others^a^	17 (10.9)	57 (15.7)	
Initial rhythm			0.68
Sinus rhythm	84 (53.9)	209 (57.4)	
Atrial tachyarrhythmias	34 (21.8)	83 (22.8)	
Ventricular tachyarrhythmias	3 (1.9)	6 (1.7)	
Other^b^	35 (22.4)	66 (18.1)	
Echocardiogram			
LV Ejection fraction, %	44.4 (27.1–57.0)	39.0 (29.0–54.5)	0.32
LV end-diastolic diameter, mm	53 (48–58)	52 (47–58)	0.52
E/e’	14.0 (9.7–20.0)	14.6 (10.9–19.6)	0.89
LA volume, mL	42.3 (29.4–60.9)	46.6 (34.1–65.4)	0.18
TAPSE, mm	12.3 (10.7–15.3)	12.4 (9.2–15.0)	0.22
RVSP, mmHg	33.6 (27.2–46.0)	36.0 (29.0–45.0)	0.63
Treatment during CICU stay			
Vasoactive drugs			
Use of additional drug^c^	92 (59.0)	123 (33.8)	<0.001
Dopamine	-	41 (11.3)	-
Norepinephrine	88 (56.4)	-	-
Epinephrine	21 (13.5)	45 (12.4)	0.73
Vasopressin	14 (9.0)	67 (18.4)	0.007
Total duration of vasoactive drug, hours	46.2 (17.1–117.3)	54.4 (20.2–177.5)	0.13
Intra-aortic balloon pump	26 (16.7)	36 (9.9)	0.03
Extracorporeal membrane oxygenation	39 (25.0)	123 (33.8)	0.05
Mechanical ventilation	84 (53.9)	264 (72.5)	<0.001
Renal replacement therapy	46 (29.5)	138 (37.9)	0.07

Values are median with interquartile range or n (%).

^a^Others include arrhythmia, pulmonary hypertension, pericardial disease, infective endocarditis, cardiac tumor, and congenital heart disease.

^b^Other includes permanent pacemaker with paced rhythm.

^c^Additional drug refers to all vasoactive drugs used after the first vasopressor regardless of the numbers of drugs used.

CICU = cardiac intensive care unit, LA = left atrium, LV = left ventricle, NT-proBNP = N-terminal pro-B-type natriuretic peptide, RV = right ventricle, RVSP = right ventricular systolic pressure, SOFA = sequential organ failure assessment, and TAPSE = tricuspid annular plane systolic excursion.

### In-hospital management

**Fig 1:**
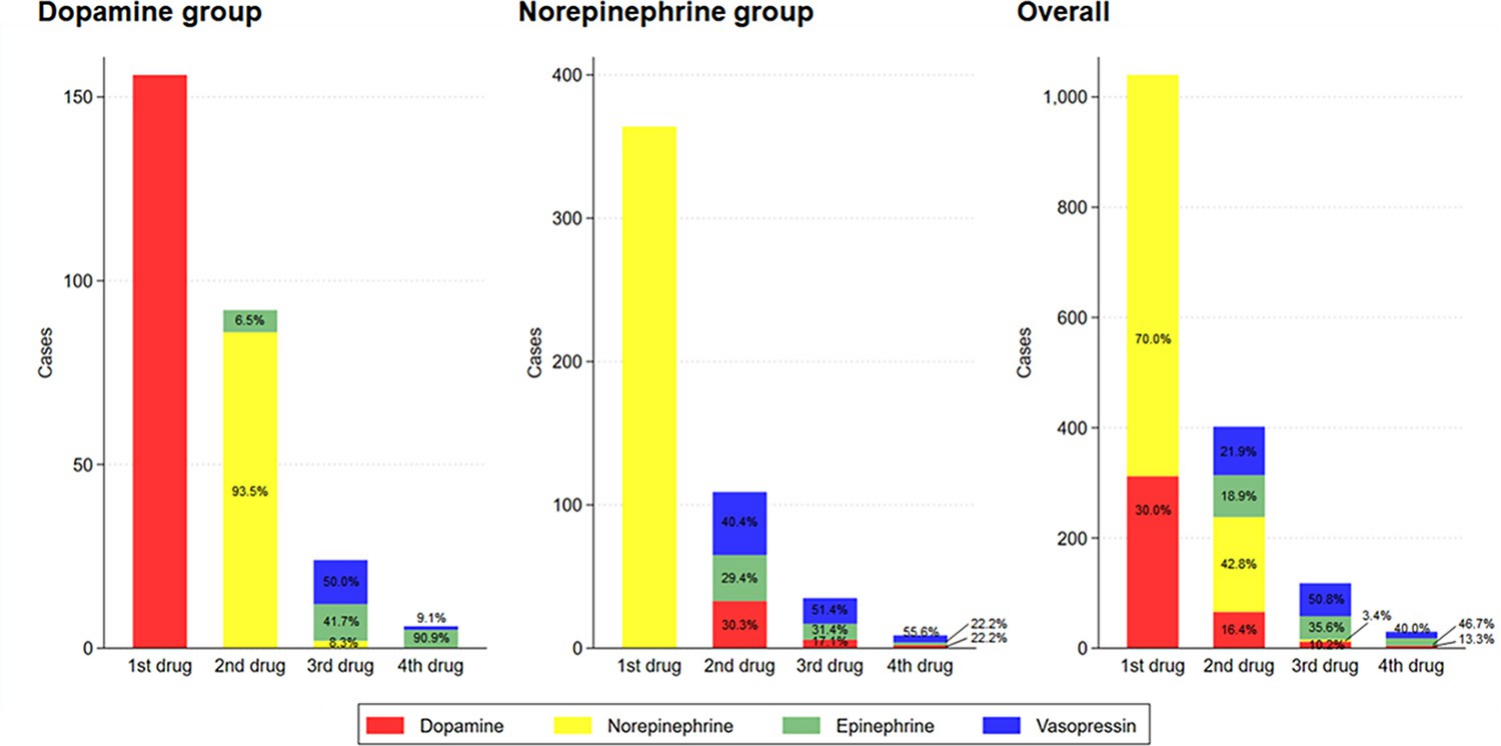
Total vasoactive drugs according to the order of use.

The total duration of vasoactive drug administration was 46.2 hours in the dopamine group and 54.4 hours in the norepinephrine group (p = 0.13) (Table 1). The dopamine group required additional vasoactive agents more frequently than the norepinephrine group (59.0% vs. 33.8%, p < 0.001). When a second-line drug was required, most patients in the dopamine group received norepinephrine (93.5%), while the patients in the norepinephrine group most often received vasopressin (35.8%), followed by dopamine (26.8%) and epinephrine (26.0%) (Fig 1). The proportion of patients requiring IABP was higher in the dopamine group (16.7% vs. 9.9%, p = 0.03), but the proportions of patients requiring ECMO (33.8% vs. 25.0%, p = 0.05), and mechanical ventilation (72.5% vs. 53.9%, p < 0.001) were higher in norepinephrine group.

### Hemodynamic changes during the initial 48 hours

**Fig 2:**
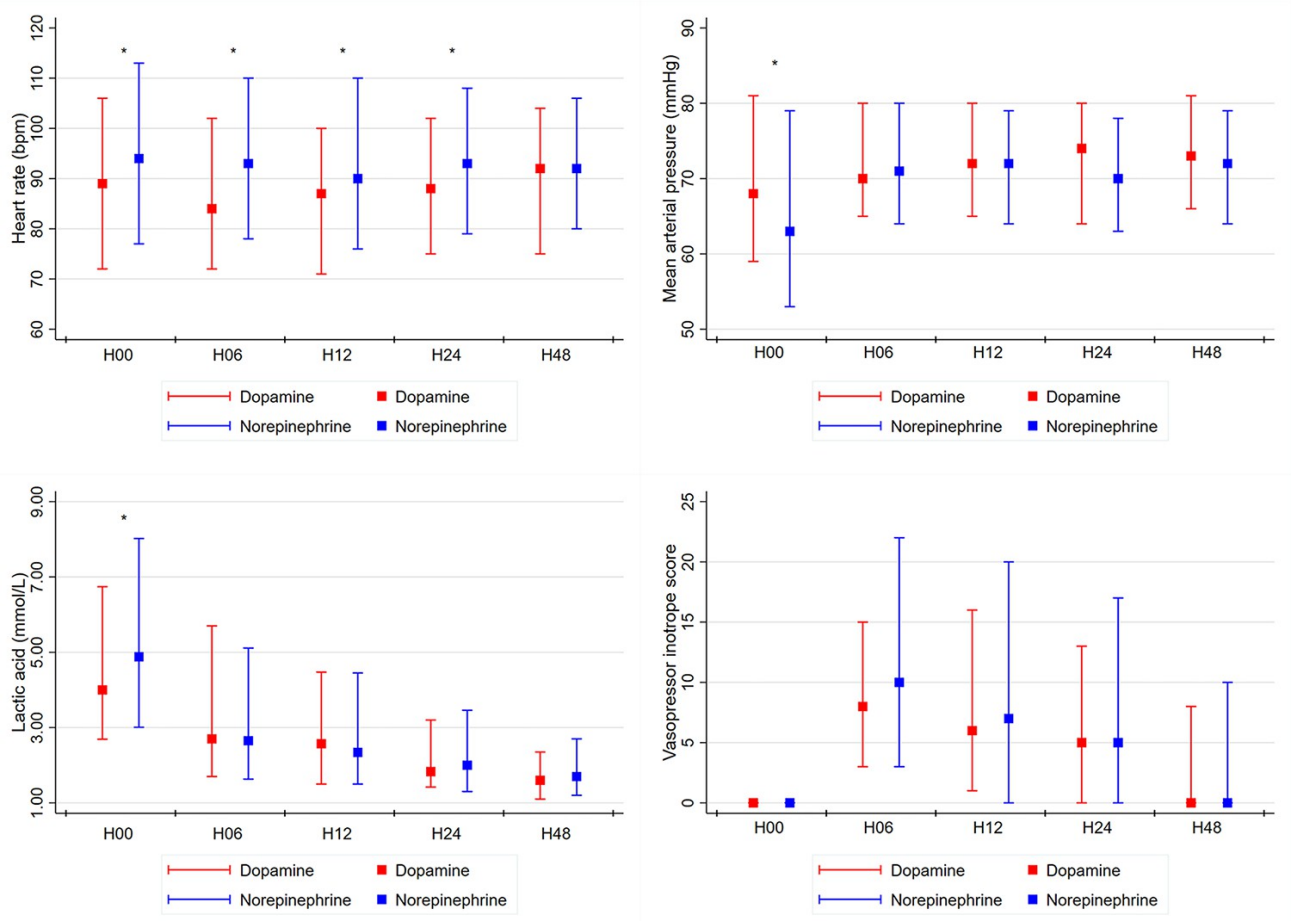
Hemodynamic parameters, lactic acid, and vasopressor inotrope score at the time of vasopressor initiation and at H6, H12, H24, and H48. Red and blue plots represent median and interquartile ranges of (A) heart rate, (B) mean arterial pressure, (C) lactic acid, and (D) vasopressor inotrope score in dopamine and norepinephrine group, respectively. *p < 0.05.

Compared to the dopamine group, the norepinephrine group had lower mean arterial pressure (68 mmHg vs. 63 mmHg, p = 0.007) and higher heart rate (89 beats/min vs. 94 beats/min, p = 0.04) and lactic acid (4.00 mmol/L vs. 4.88 mmol/L, p = 0.02) at the initial phase (H_0_) (Fig 2).

Heart rate was higher in the norepinephrine group until H_24_, but the difference between groups for mean arterial pressure (H_6_: 70 mmHg vs. 71 mmHg, p = 0.98; H_12_: 72 mmHg vs. 72 mmHg, p = 0.37; H_24_: 74 mmHg vs. 70 mmHg, p = 0.06; H_48_: 73 mmHg vs. 72 mmHg, p = 0.45) and lactic acid (H_6_: 2.70 mmol/L vs. 2.65 mmol/L, p = 0.55; H_12_: 2.57 mmol/L vs. 2.34 mmol/L, p = 0.71; H_24_: 1.83 mmol/L vs. 2.00 mmol/L, p = 0.72; H_48_: 1.60 mmol/L vs. 1.70 mmol/L, p = 0.32) was no longer significant after H6. VIS showed no significant differences between the two groups during the 48 hours follow-up.

### Clinical outcomes

Overall, 158 (30.4%) patients died while hospitalized, and 136 (26.2%) of these patients died in the CICU. There were no differences between the two groups in in-hospital mortality (26.9% vs. 31.9%, p = 0.26) and CICU mortality (25.6% vs. 26.4%, p = 0.86). Atrial fibrillation and ventricular tachyarrhythmia were newly identified in 14.6% and 23.7% of patients, respectively, and the incidence of arrhythmia was similar in both groups. The groups did not differ in occurrence of cerebrovascular events, bleeding, or sepsis. Mortality and incidence of arrhythmia, cerebrovascular events, bleeding, and sepsis were similar in propensity score-matched patients (Table 2).

**Table 2:** Clinical outcomes.

Variables	Dopamine group (n = 156)	Norepinephrine group (n = 364)	p-value
In-hospital mortality	42 (26.9)	116 (31.9)	0.26
Cardiovascular cause	27 (64.3)	77 (66.4)	
Non-cardiovascular cause	15 (35.7)	39 (33.6)	
Complications			
Arrhythmia			
Atrial fibrillation	19 (12.2)	57 (15.7)	0.30
Ventricular tachyarrhythmia	31 (19.9)	92 (25.3)	0.18
Cerebrovascular event	1 (0.6)	4 (1.1)	>0.99
Bleeding	2 (1.3)	5 (1.4)	>0.99
Sepsis	7 (4.5)	18 (5.0)	0.82

Values are median with interquartile range or n (%).

Logistic regression analysis identified no significant differences in in-hospital mortality between the dopamine group and norepinephrine group among total patients or propensity score-matched patients, regardless of adjustment for clinically relevant variables (S2 Fig). The differences in effects of dopamine and norepinephrine were not significant across subgroups and there were no significant interactions between treatment effect and any of the subgroups with respect to in-hospital mortality (S3 Fig).

## Discussion

In this study, we investigated the practice pattern for administration of additional vasopressors and hemodynamic changes in CS patients treated with dopamine or norepinephrine as the first-line vasopressor and evaluated whether the selection of first-line vasopressor was associated with clinical outcomes. We demonstrated that the use of norepinephrine as the first-line vasopressor did not reduce in-hospital or CICU mortality, or arrhythmia, in patients with CS compared to the use of dopamine. In addition, these findings were consistent across subgroups and in propensity-matched samples. Furthermore, mean arterial pressure and lactate level were not significantly different between groups 6 hours after starting vasopressors. However, additional vasopressors were required in a substantial number of patients treated with dopamine as the first-line vasopressor.

Norepinephrine was more commonly used as the first-line vasopressor and was preferred in patients with lower blood pressure and higher heart rates at the time of initiation of vasopressor in this study. This practice reflects relevant guidelines. The 2004 American College of Cardiology/the AHA guidelines recommend vasopressor support for hypotension that does not recover after volume loading, and indicate dopamine as the agent of first choice [9]. However, administration of norepinephrine, a more potent vasoconstrictor than dopamine, is suggested in markedly hypotensive cases with systolic blood pressure < 70mmHg. In 2010, the Sepsis Occurrence in Acutely Ill Patients (SOAP) II trial, a randomized trial comparing the use of dopamine and norepinephrine in patients with shock, showed that the rate of death at 28 days was significantly higher among patients with pre-specified CS subgroup (n = 280) who were treated with dopamine than those treated with norepinephrine [16]. In particular, patients treated with dopamine had higher incidence of arrhythmia than those treated with norepinephrine in the entire study population, and this was considered an advantage of norepinephrine. Although the SOAP II trial is the largest study focusing on vasopressor selection in shock patients, only 135 patients treated with dopamine and 145 patients treated with norepinephrine were included and adequate external validation was not performed. Consequently, there is insufficient evidence to date from well-controlled randomized trials that norepinephrine is superior to dopamine in CS patients. The AHA guidelines for management of CS published in 2017 noted that the first-line vasopressor should be individually selected according to heart rate or accompanying risk of arrhythmia, although norepinephrine is associated with fewer arrhythmias and may be the vasopressor of choice in many patients [1, 17]. The practice pattern of vasopressor selection shown in our study, in that dopamine was selected for patients with lower heart rates and norepinephrine was selected for patients with lower mean arterial pressure, reflects the individualized approach.

However, subgroup analysis according to heart rate or mean arterial pressure did not indicate superiority of any specific vasopressor. Considering that an additional vasopressor, usually norepinephrine, was required in a substantial number of patients treated with dopamine, it is possible that the cumulative effect of multiple vasopressors was similar between the two groups. This may be why there were no differences in hemodynamic parameters except for heart rate from 6 hours after vasopressor initiation and clinical outcomes between the two groups were similar. Generally, multiple vasopressors are used when adequate hemodynamic status cannot be achieved with a single vasopressor for the management of CS in real practice. Therefore, although we did not identify differences in clinical outcomes according to the first-line vasopressor in this study, the higher rate of additional vasopressor uses in the dopamine group than norepinephrine group may indirectly support current guidelines recommending norepinephrine as the first-line vasopressor in CS.

Although vasopressor therapy plays a key role in maintaining hemodynamic status in the treatment of patients with CS, identifying and treating the underlying etiology that caused CS is an essential part of the management of CS [1, 2]. In particular, early revascularization is associated with improvement of clinical outcomes in CS complicating acute coronary syndrome [3, 18]. About half of the patients included in our study were CS with acute coronary syndrome, and revascularization was performed in most of these patients. The use of mechanical circulatory support devices is a means of maintaining adequate tissue perfusion in patients with shock refractory to vasopressor therapy [19]. Although mechanical circulatory support is complex and is associated with several complications, timely initiation in patients with refractory CS can improve survival [20, 21]. Refractory to medical therapy alone is common in the management of CS, and about 40% of the patients in our study required IABP or ECMO for circulatory support. Given that about 20% of patients in the SOAP II trial died from refractory shock, we cannot rule out the possibility that the use of mechanical circulatory support devices influenced the results of our study [16].

Although this study provides additional information on the association of first-line vasopressor selection with mortality in patients with CS, several limitations should be noted. First, our study was retrospective and observational in nature. Therefore, although patient management was performed according to general guidelines, there were no protocols for dose adjustment, combination and discontinuation of vasoactive drugs, target blood pressure, or timing of mechanical circulatory support. Thus, selection bias and confounding may have affected our results. However, we performed regression modeling and subgroup analysis including various risk factors to control for confounders. Next, because our study was conducted in a single tertiary care center with a CICU that satisfies level 1 requirements, the generalizability of our findings to other centers with different staffing, monitoring and therapeutic technologies may be limited. Finally, we did not include hemodynamic criteria such as cardiac index or pulmonary artery wedge pressure in defining CS. However, routine use of pulmonary artery catheters is gradually decreasing in real practice [22]. In addition, several previous studies used criteria similar to ours [4, 23]. It will be necessary to reach consensus on a unified and pragmatic definition of CS, such as the recently used SCAI classification, to improve the treatment of patients with CS [24].

## Conclusion

The use of norepinephrine as a first-line vasopressor was not associated with reduction of in-hospital mortality or early recovery of hemodynamic parameters but could reduce use of additional vasopressors compared to use of dopamine in CS patients. Further randomized controlled studies with larger numbers of patients will be required to confirm our findings.

## Supporting information

S1 Table Clinical characteristics and outcomes after propensity score matching. (DOCX)

S1 Fig Scheme of group distribution. (DOCX)

S2 Fig Effect of norepinephrine on hospital mortality in cardiogenic shock. (DOCX)

S3 Fig Forest plot for subgroup analysis comparing dopamine with norepinephrine. (DOCX)

## Abbreviations

AHA: American Heart Association
CICU: Cardiac intensive care unit
CRRT: Continuous renal replacement therapy
CS: Cardiogenic shock
ECMO: Extracorporeal membrane oxygenation
IABP: Intra-aortic balloon pump
SOAP: Sepsis Occurrence in Acutely Ill Patients study
SOFA: Sequential organ failure assessment
VIS: Vasopressor Inotrope Score
