# Supplementary material for: Dopamine versus norepinephrine as the first-line vasopressor in the treatment of cardiogenic shock
Source: PLoS One. 2022 Nov 3;17(11):e0277087. doi: 10.1371/journal.pone.0277087 (PMC9632770; doi:10.1371/journal.pone.0277087)
Supplement: S1 Table — (DOCX) [file pone.0277087.s001.docx]

**Supporting information**

**S1 Table. Clinical characteristics and outcomes after propensity score matching**

| Variables | Dopamine group  (n = 140) | Norepinephrine group  (n = 140) | *P*-value |
| --- | --- | --- | --- |
| Age, years | 66 (56-77) | 69 (60-77) | 0.26 |
| Male | 85 (60.7) | 86 (61.4) | 0.90 |
| SOFA score | 7 (5-10) | 8 (5-10) | 0.88 |
| Cardiac arrest prior to admission | 32 (22.9) | 33 (23.6) | 0.89 |
| Comorbidities |  |  |  |
| Diabetes | 54 (38.57) | 62 (44.3) | 0.33 |
| Hypertension | 71 (50.71) | 83 (59.3) | 0.15 |
| Coronary artery disease | 39 (27.86) | 59 (42.1) | 0.01 |
| Chronic heart failure | 42 (30) | 45 (32.1) | 0.70 |
| Chronic lung disease | 8 (5.71) | 5 (3.6) | 0.39 |
| Chronic kidney disease | 30 (21.43) | 37 (26.4) | 0.33 |
| Chronic liver disease | 6 (4.29) | 9 (6.4) | 0.43 |
| Diagnosis |  |  | 0.88 |
| Acute coronary syndrome | 79 (56.43) | 77 (55.0) |  |
| Heart failure | 48 (34.29) | 47 (33.6) |  |
| Others^*^ | 13 (9.29) | 16 (11.4) |  |
| Initial rhythm |  |  | 0.44 |
| Sinus rhythm | 79 (56.4) | 85 (60.7) |  |
| Atrial tachyarrhythmias | 29 (20.7) | 27 (19.3) |  |
| Ventricular tachyarrhythmias | 3 (2.1) | 0 (0.0) |  |
| Other^†^ | 29 (20.7) | 28 (20.0) |  |
| Mean arterial pressure, mmHg | 68 (60-82) | 69 (58-88) | 0.92 |
| Heart rate, beat/min | 92 (74-106) | 89 (75-107) | 0.92 |
| Laboratory tests |  |  |  |
| Lactic acid, mmol/L | 4.24 (2.86-6.94) | 4.43 (2.88-7.57) | 0.48 |
| Troponin I, ng/mL | 16.36 (0.52-97.35) | 16.74 (0.73-83.03) | 0.87 |
| NT-proBNP, pg/mL | 6541 (573-21657) | 8512 (2091-33599) | 0.05 |
| LV Ejection fraction, % | 41.3 (27.1-57.0) | 40.0 (25.1-50.3) | 0.30 |
| Treatment during CICU stay |  |  |  |
| Total duration of vasoactive drug use, hours | 46.2 (17.5-117.4) | 46.7 (18.2-159.3) | 0.48 |
| Intra-aortic balloon pump | 24 (17.1) | 15 (10.7) | 0.12 |
| Extracorporeal membrane oxygenation | 36 (25.7) | 41 (29.3) | 0.50 |
| Mechanical ventilation | 78 (55.7) | 89 (63.6) | 0.18 |
| Renal replacement therapy | 39 (27.9) | 52 (37.1) | 0.22 |
| Clinical outcomes |  |  |  |
| Hospital mortality | 38 (27.1) | 31 (22.1) | 0.33 |
| Cause of death |  |  | 0.73 |
| Cardiovascular | 26 (68.4) | 20 (64.5) |  |
| Non-cardiovascular | 12 (31.6) | 11 (35.5) |  |
| Complications |  |  |  |
| Arrhythmia |  |  |  |
| Atrial fibrillation | 17 (12.1) | 27 (19.3) | 0.10 |
| Ventricular tachyarrhythmia | 30 (21.4) | 32 (22.9) | 0.77 |
| Cerebrovascular event | 1 (0.7) | 2 (1.4) | 0.50 |
| Bleeding | 2 (1.4) | 3 (2.1) | 0.62 |
| Sepsis | 7 (5.0) | 4 (2.9) | 0.36 |

Values are median with interquartile range or n (%).

*. Others include arrhythmia, pulmonary hypertension, pericardial disease, infective endocarditis, cardiac tumor, and congenital heart disease.

^†^. Other includes permanent pacemaker with paced rhythm.

CICU = cardiac intensive care unit, LV = left ventricle, NT-proBNP = N-terminal pro-B-type natriuretic peptide, and SOFA = sequential organ failure assessment.
