# Supplementary material for: Dopamine versus norepinephrine as the first-line vasopressor in the treatment of cardiogenic shock
Source: PLoS One. 2022 Nov 3;17(11):e0277087. doi: 10.1371/journal.pone.0277087 (PMC9632770; doi:10.1371/journal.pone.0277087)
Supplement: S1 Fig — (DOCX) [file pone.0277087.s002.docx]

**Supporting information**

**S1 Fig. Scheme of group distribution.**
